# Ischemic infarct detection, localization, and segmentation in noncontrast CT human brain scans: review of automated methods

**DOI:** 10.7717/peerj.10444

**Published:** 2020-12-18

**Authors:** Wieslaw L. Nowinski, Jerzy Walecki, Gabriela Półtorak-Szymczak, Katarzyna Sklinda, Bartosz Mruk

**Affiliations:** 1 John Paul II Center for Virtual Anatomy and Surgical Simulation, University of Cardinal Stefan Wyszynski, Warsaw, Poland; 2 Department of Radiology and Diagnostic Imaging, Center of Postgraduate Medical Education, Warsaw, Poland

**Keywords:** Ischemic stroke, Human brain, Detection, Localization, Segmentation, Noncontrast CT, Brain atlas, Image processing, Image analysis, Artificial intelligence, Review

## Abstract

Noncontrast Computed Tomography (NCCT) of the brain has been the first-line diagnosis for emergency evaluation of acute stroke, so a rapid and automated detection, localization, and/or segmentation of ischemic lesions is of great importance. We provide the state-of-the-art review of methods for automated detection, localization, and/or segmentation of ischemic lesions on NCCT in human brain scans along with their comparison, evaluation, and classification. Twenty-two methods are (1) reviewed and evaluated; (2) grouped into image processing and analysis-based methods (11 methods), brain atlas-based methods (two methods), intensity template-based methods (1 method), Stroke Imaging Marker-based methods (two methods), and Artificial Intelligence-based methods (six methods); and (3) properties of these groups of methods are characterized. A new method classification scheme is proposed as a 2 × 2 matrix with local versus global processing and analysis, and density versus spatial sampling. Future studies are necessary to develop more efficient methods directed toward deep learning methods as well as combining the global methods with a high sampling both in space and density for the merged radiologic and neurologic data.

## Introduction

Stroke is a major reason for permanent disability and a leading cause of death, which affects public health and results in large costs (Katan & Luft, 2018). Diagnostic imaging plays a central role in stroke management with Computed Tomography (CT) and/or Magnetic Resonance (MR) imaging employed to make the diagnosis and therapeutic decisions. Noncontrast Computed Tomography (NCCT) remains the first-line diagnosis for emergency evaluation of acute stroke because it is fast, widely available, cost-efficient, and reliably rules out hemorrhage (Lövblad & Baird, 2010). Early ischemic changes in NCCT scans are characterized by several features including the presence of hypodensity within the infarcted region, loss of distinction between gray and white matter, diminishing of the basal ganglia contrast, sulcal effacement, ventricular narrowing, disappearing of insular ribbon, and/or a middle cerebral artery hyperdensity sign. We also have observed the presence of the hyperdense posterior cerebral artery sign (Ambrosius et al., 2011). NCCT, however, has poor sensitivity, particularly in the first few hours, as acute ischemic changes on NCCT are subtle and often do not show infarct until 12–24 h after stroke onset (James et al., 2006). When compared to MR, this sensitivity is 25% in NCCT versus 86% in MR; however, within the first 3 h, it is lowered to 7% for NCCT and 46% for MR (Chalela et al., 2007).

The stroke-caused changes in a NCCT image may be too imperceptible to the human eye to be detected, particularly in the hyperacute stage, therefore their computer-assisted processing and analysis could assist in enhancing and expediting the scan reading. In general, there are three approaches to delineate lesions in brain images: manual, semi-automated, and fully automated, and a manual tracing by trained professionals remains the gold standard (Fiez, Damasio & Grabowski, 2000; Wilke et al., 2011). Especially for follow-up scans, the reliable and reproducible lesion segmentation is of high interest, as the lesion volume is one important imaging end-point for clinical trials. A manual delineation of a brain lesion is laborious and time-consuming, and it requires substantially more human input compared to the automated approach (e.g., a few hours versus one minute as compared by Wilke et al. (2011)). Therefore, automating the detection and segmentation of ischemic infarcts is critical, especially, as the time window to treat stroke is 3-4.5 h for intravenous thrombolysis (Hacke et al., 2008). Moreover, a manual approach results in variability across operators, because there is often no clear cutoff between lesioned and non-lesioned tissues, especially, at the brain’s borders and around the cerebral ventricles as well as it usually does not detect inevitable stroke-induced changes taking place outside the lesion (Fiez, Damasio & Grabowski, 2000). Semi-automated methods combine advantages of a fully automated abnormality detection with manual editing of the lesion enabling the operator to finalize its location and extent.

A computer-assisted approach, even without applying fully automated methods, has been employed in stroke image management. For instance, Mainali, Wahba & Elijovich (2014) demonstrated that a simple transformation of image brightness and contrast, by changing the window center and width level in standard windows and introducing improved stroke windows, significantly improves detection (from 18% to 70%) of early ischemic changes.

A popular approach for stroke detection is to apply the ASPECTS score. The ASPECTS (meaning the Alberta Stroke Program Early CT Score) aims to systemize the detection and reporting of ischemic stroke by visually identifying an ischemic hypodensity on the middle cerebral artery (MCA) territory subdivided into ten regions that are located on two different axial CT slices (Barber et al., 2000). In order to automate this visual approach, Kuang et al. (2019) proposed a method based on texture features extracted from each ASPECTS region to train a random forest classifier. This automated approach tested on 100 patients showed a reasonable ability to determine the ASPECTS.

Numerous methods have been developed for semi-automated and automated stroke lesion detection and delineation, mostly in MR images; however, only a few approaches have been proposed for detecting stroke lesions in NCCT scans (Rekik et al., 2012). Moreover, in comparison to the development of hemorrhagic stroke processing methods that of ischemic stroke detection is given less attention because of its more demanding nature (Gillebert, Humphreys & Mantini, 2014).

Besides the speed and operator independence, there are several additional advantages of employing a computer-assisted fully automated infarct detection and localization in NCCT as discussed by Nowinski et al. (2013). Namely, first, the computer is able to process multiple density ranges in order to detect subtle changes in them. Second, the density changes caused by infarction can be accumulated across all slices in 3D by the computer, which may facilitate the detection and localization of these changes. Third, the computer is able to identify and compare contralateral regions (i.e., symmetrical with respect to the calculated midsagittal plane) which symmetry is critical, particularly, when the symmetry in the original images is deteriorated or even completely vanished due to a heavy head tilt (which often happens in scans acquired in the emergency room).

Though there exist several methods aiming to automate the evaluation of ischemic stroke in MR and CT images, only a few of them address the automated detection, localization, and/or segmentation of ischemic lesions in NCCT human brain scans. Rekik et al. (2012) presented a state-of-the-art review in the medical image analysis approaches applied to segmentation, prediction, and dynamic evolution modeling of acute/subacute ischemic stroke from CT and MR human, animal and/or synthetic data. Out of 44 papers included in that review, in the category of automated segmentation of human ischemic stroke from NCCT the review lists only six papers from four centers. These papers are categorized into three groups: image-based methods (Matesin, Loncaric & Petravic, 2001; Meilunas et al., 2003; Usinskas et al., 2003; Usinskas, Dobrovolskis & Tomandl, 2004), pixel-based methods (Chawla et al., 2009), and atlas-based methods (Maldijan et al., 2001).

Another review prepared by Mokli et al. (2019) provides a computer-aided imaging analysis in acute ischemic stroke from NCCT, computed tomography angiography, and perfusion imaging. This review lists 26 software applications commercially available from 13 companies for automated and semi-automated medical image analysis for acute stroke diagnostics, and only two of them deal with NCCT ischemic stroke, each employing the ASPECTS score. These are e-ASPECTS^®^ software from Brainomix Ltd. (Oxford, UK) to assess the ASPECTS score and volume of ischemia; and RAPID ASPECTS^®^ from iSchemaView (Menlo Park, USA) to automatically identify and score regions with early ischemic changes using the ASPECTS score. Up to date, these are the only two commercial products available that are certified for use in clinical routine. In addition, Frontier ASPECTS from Siemens Healthcare GmbH (Erlangen) is another ASPECTS-based application developed that is not yet certified for clinical application.

The goal of this work is to provide: (1) review of the state-of-the-art methods for automated detection, localization, and/or segmentation of ischemic lesions in human brain NCCT scans, (2) comparison, evaluation, and classification of the reviewed methods, and (3) recommendation for future developments.

### Survey methodology

We searched the literature in the examined scope of interest using PubMed and Google Scholar, scanning also in the corresponding references, selected papers, and related articles. We employed the following keywords: “stroke”, “ischemic stroke”, automated detection”, “automated segmentation”, “automated localization”, and “noncontrast CT”.

A number of various methods have been proposed for automated detection, localization, and/or segmentation of ischemic lesions on NCCT in human brain scans. We classify these methods into five groups:

1. Image processing and analysis-based methods;
2. Brain atlas-based methods;
3. Intensity template-based methods;
4. Stroke Imaging Marker (SIM)-based methods;
5. Artificial Intelligence (AI)-based methods.

### Image processing and analysis-based methods

Several image processing and analysis-based methods have been proposed for automated handling of NCCT stroke images by employing a variety of techniques including, among others, thresholding, region growing, edge detection, textures, wavelets, rule-based expert systems, classification, and combination of them.

Matesin, Loncaric & Petravic (2001) proposed a rule-based method for segmentation and labeling of ischemic stroke lesions. The method consists of three steps as previously described in Matesin, Loncaric & Petravic (2001): determination of a head symmetry axis based on moments, seeded region growing to identify multiple regions having uniform brightness, and rule-based region labeling by using an expert system. The rules for identifying the background, skull, brain, and cerebrospinal fluid (CSF) are neighborhood- and intensity-based, and those for an ischemic lesion are symmetry-based. The authors claimed feasibility without presenting any quantitative results.

The group by Meilunas and Usinskas presented three works. Meilunas et al. (2003) proposed a method based on the contouring of an ischemic stroke region boundary. The method consists of slice filtering, smoothing, and extension of stroke region boundary followed by the computing of an infarct volume.

Usinskas et al. (2003) compared a few methods suggesting that the best viability for ischemic stroke area segmentation showed mean, standard deviation, histogram, and gray level co-occurrence matrix methods as well as a supervised artificial neural network technique.

Subsequently, Usinskas, Dobrovolskis & Tomandl (2004) proposed a texture-based method with an unsupervised classifier. The method uses 18 unified textural features to segment an ischemic stroke region on images, including joint features from the mean, standard deviation, histogram, and gray level co-occurrence matrix. The method requires thresholding for each image which is not automated. The authors showed an ability to segment an ischemic stroke region without any quantitative assessment.

Przelaskowski et al. (2007) proposed a wavelet-based method. The authors observed that infarction perception can be improved by data denoising and local contrast enhancement in a multi-scale domain, and presented a wavelet-based image processing method enhancing the subtlest signs of hypodensity which were often invisible in a standard CT scan review. The method studied on 30 ischemic scans increased the sensitivity of ischemic lesion detection from 12.5% for a standard CT scan preview to 56.3%.

Chawla et al. (2009) proposed a classification-based method in the intensity and wavelet domains. The method detects and classifies a stroke-related abnormality into acute infarct, chronic infarct, and hemorrhage by comparing the cerebral hemispheres. The method consists of three steps as previously reported in Chawla et al. (2009): image enhancement and denoising, detection of a brain symmetry line, and classification of abnormal slices. A two-level classification scheme employs an intensity histogram-based comparison to identify chronic and hemorrhagic cases as well as wavelet energy-based texture information for acute infarct detection. The method was evaluated on 6 normal and 9 stroke patient CT scans resulting in an accuracy of 90%.

Tang, Ng & Chow (2011) and Tang, Ng & Chow (2013) proposed a texture-based method using circular adaptive regions of interest. This method comprises preprocessing (a threshold-based bone and artifact removal), generation of circular adaptive regions of interest, for each region locating by reflection a corresponding circular region on the other side of the brain image, and comparing each pair of the circular regions with several texture attributes. These attributes are calculated based on a gray level co-occurrence matrix and they include energy, entropy, inverse difference moment, inertia, prominence, shade, correlation, and variance. The method was tested on 10 acute and 10 chronic ischemic stroke cases resulting in an estimated accuracy of 86.96%.

Boers et al. (2013) presented a method for infarct volume measurement in follow-up NCCT scans by employing region growing. After a manual placement of the seed point in the infarcted hypo-attenuated area, the region growing was repeated for various thresholds in the range of 1.5–4.5 HU (Hounsfield Units) with a step of 0.5 HU resulting in seven segmentations. To avoid the region growing from leaking into the contralateral hemisphere, the midline was used as a limit (that was determined based on the geometric center and the most extreme midsagittal bone or nasal cartilage structures). The algorithm was tested on 34 cases and achieved the Dice’s Similarity Coefficient (DSC) (Dice, 1945) of 34%.

Vos et al. (2013) proposed a classification-based method to detect and segment ischemic lesions. The method comprises three stages as previously described in Vos et al. (2013): pixel classification and lesion candidate localization (via a naive Bayes classifier combined with a tissue homogeneity processing to localize candidates for ischemic lesions), segmentation of the candidate lesions and feature extraction (through a marching cubes algorithm to analyze regional statistics in order to extract features based on local and contextual information from the contralateral hemisphere), and aggregation of the extracted features into a likelihood of ischemia (by employing a supervised classifier). The method performance in lesion segmentation achieved the DSC of 74%.

Tyan et al. (2014) presented an unsupervised feature perception enhancement method for ischemic stroke detection. The method works in four-steps as previously reported in Tyan et al. (2014): preprocessing (utilizing a cubic curve contrast enhancement), brain tissue extraction (by applying thresholding, blurring, and morphological operations), meaningful area extraction (through edge detection to identify the stroke area and unsupervised region growing followed by a brain area partitioning into eight regions by a horizontal and a vertical line and an elliptic curve determining the border between the gray matter (GM) and white matter (WM)), and infarct regional location (by calculating the brightness in these eight regions, determining areas with the smallest values in comparison to their counterparts, and analyzing their mutual relationships). The method tested on 26 patients demonstrated an increased stroke diagnosis sensitivity of 83% in comparison to 31% when radiologists used conventional diagnostic images.

Ray & Bandyopadhyay (2016) proposed a method based on the textural analysis. The method contains three modules as previously featured in Ray & Bandyopadhyay (2016): preprocessing (by performing noise and artifacts removal), segmentation (by applying image gradient magnitude watersheding followed by thresholding), and feature extraction (by dividing an image into four quadrants, calculating for each region first-order texture measures (the mean, standard deviation, variation, skewness, kurtosis, and entropy), and selecting an abnormal region based on their values). The results contain a single image of an extracted hemorrhage, without any ischemic lesions and quantitative assessment.

### Brain atlas-based methods

A brain atlas is a means for knowledge aggregation, presentation, and discovery (Nowinski, 2020a). Electronic brain atlases have potential usefulness in stroke image management for diagnosis, treatment, and prediction (Nowinski, 2020b). In ischemic stroke detection, a brain atlas enables an automated generation of regions of interest (ROIs).

Maldijan et al. (2001) presented an atlas-enhanced method for identifying potential areas of acute ischemia for middle cerebral artery (MCA) stroke. The method first performs image preprocessing, including interpolation, scalp striping, normalization, and atlas-based segmentation of the lentiform nucleus and insula. Then, voxels densities in the segmented lentiform nucleus and insula of one hemisphere are compared with those in the contralateral side by using the Wilcoxon two-sample rank-sum test. This method, limited to two structures and MCA ischemic stroke, was validated for 15 ischemic stroke patients. Note that conceptually, the method is similar to the ASPECTS scale with a lower number of ROIs, though delineated and processed automatically.

Nowinski (2020b) proposed a multi-atlas method to detect and localize ischemic and hemorrhagic stroke lesions. It processes the entire brain covered with numerous ROIs that are examined, overcoming the limitation of the ASPECTS-based and Maldijan et al. (2001) methods regarding a small number of ROIs. These ROIs are derived from two atlases, an atlas of anatomy and an atlas of blood supply territories, which are in spatial correspondence (Nowinski et al., 2006b). As previously described in Nowinski (2020b) the method calculates the midsagittal plane separating the brain into the right and left hemispheres and is able to handle a large head tilt often present in emergency room acquisitions (Puspitasari et al., 2009), maps the brain atlases on an NCCT scan through the ellipse-fitting atlas-to-scan registration method (Volkau et al., 2012), extracts the ventricular system using a dedicated algorithm for segmentation of the ventricular system from ischemic stroke NCCT scans (Poh et al., 2012) and removes from the processed images the CSF regions as the CSF density range overlaps with that of infarcts, determines numerous left–right corresponding ROIs in both hemispheres by employing the atlases, and compares these pairs of the ROIs by means of multiple statistical tests. The method was tested on several ischemic and hemorrhagic cases demonstrating feasibility, and the component algorithms were validated quantitatively on 208 NCCT scans (MSP extraction), 75 multi-modal NCCT, MR, and PET scans (atlas-to-scan mapping), and 102 NCCT stoke cases (ventricular system extraction).

### Intensity template-based methods

Gillebert, Humphreys & Mantini (2014) proposed an intensity template-based method to delineate infarcts and hemorrhages. The method comprises two steps as previously described in Gillebert, Humphreys & Mantini (2014): patient scan preprocessing (by applying a threshold-based clustering, intensity transformation, MNI (Montreal Neurological Institute) space normalization, isotropic reslicing, and smoothing) followed by statistical analysis for lesion detection (by a voxel-by-voxel comparison of the preprocessed scan with a normal CT scan template developed from 72 non-stroke subjects for defining areas with hypo- or hyper-intense signals). The performance measured by the DSC on 24 acute stroke patients and 72 control subjects with the simulated lesions ranged (depending on the degree of applied smoothing and the level of thresholding) between 52% and 89%.

### SIM-based methods

The image processing and analysis-based methods typically detect local changes in images, while the SIM-based methods attempt to capture global density changes caused by an infarct in the scan. Moreover, the SIM-based methods make detection taking into account the actual patient’s values of CSF, WM, and GM. Finally, these methods avoid image processing operations distorting original densities values, such as smoothing or blurring.

### Standard SIM-based method

Nowinski et al. (2013) proposed a method for rapid and automatic detection, localization, and volume assessment of ischemic infarcts (including hyperacute, acute, lacunar, and chronic infarcts as well as infarcts with hemorrhagic transformation and leukoaraiosis). The method exploits the fact that an ischemic lesion manifests itself by (1) occupying the density range between CSF and WM, and (2) redistribution of density globally between the hemispheres. This redistribution, which might be barely observable by the human eye, is captured by the introduced Stroke Imaging Marker (SIM). The SIM determines the infarct spatial range in the axial, coronal, and sagittal orientations by statistically comparing multiple cumulative density distributions calculated for the whole normal and infarcted cerebral hemispheres. As previously described in Nowinski et al. (2013) the method performs in five main steps: (1) identify the midsagittal plane (MSP) by applying the algorithm by Puspitasari et al. (2009) and subdivide the brain into the right and left hemispheres; (2) reformat the originally acquired axial slices through near neighbor interpolation (to avoid changing the original density values) such as to be precisely perpendicular to the MSP, as typical stroke acquisitions usually do not produce exactly symmetrical images; (3) calculate for each hemisphere the patient-specific density ranges of CSF, WM, and GM employing the algorithm by Gupta et al. (2010); (4) compute the spatial extent of the ischemic infarct by determining its range in each axial, coronal, and sagittal orientations through the SIM; and (5) calculate two cuboidal regions inner and outer that localize the ischemic infarct in 3D.

The SIM is composed of three components and it is computed as:

SIM = P-ratio * N-ratio / MDV,

where:

• P-ratio denotes a percentile difference ratio in several small subranges of the entire density distribution;
• N-ratio denotes a ratio of voxels count in two density bands within the brain parenchyma;
• MDV is the median density value.

As previously explained in Nowinski et al. (2013) the SIM is computed for every image in each hemisphere, and each axial, coronal, and sagittal orientation. The SIM is adaptive to diverse manifestations of the infarcted region by employing a set of parameters determining the density subranges resulting in 54 various combinations (SIM plots). The combination that achieves the maximum SIM difference across the normal and infarcted hemispheres is taken as the most significant result by applying the Wilcoxon rank-sum test. The spatial extent of the infarct is determined by the starting and ending locations of the intersection points of the SIM plots for both hemispheres enclosing the largest number of the consecutive slices. The 5th and 95th percentiles of the distribution of the starting and ending intersections are considered the lesion localization limits, and when calculated for the axial, coronal, and sagittal orientations they demarcate two bounding boxes, the inner and the outer, that localize spatially the infarct.

As described earlier by Nowinski et al. (2013) the method was quantitatively validated on 576 clinically confirmed strokes, each with a single NCCT scan. The scans were acquired at four centers in two countries. The scans consisted of core scans of 322 “pure” acute ischemic infarcts (i.e., without any other noticeable pathology), 36 lacunar infarcts, 17 hemorrhagic transformations, 104 ischemic infarcts jointly with chronic infarcts, and 70 acute ischemic infarcts along with leukoaraiosis. Out of the total of (104+70) chronic infarcts and leukoaraiosis cases, 27 scans had ischemic infarcts along with both leukoaraiosis and chronic infarcts. The time after the onset of symptoms at acquisition was available for 532 scans, and it spanned from 1.5 h (hours) to 72 h for 450 scans and above 72 h for 82 others. These scans were divided into 3 h, 3< to 8 h, and >8 h after the onset of symptoms. In addition, 21 NCCT hyperacute cases (between 1.5 h and 7 h) with additional follow-up NCCT imaging were used for early stroke detection.

As previously reported in Nowinski et al. (2013) the SIM method matched 100% expert’s infarct detection achieving 99.8% inner localization specificity and 93.3% outer localization sensitivity when leukoaraiosis cases, chronic infarcts, and infarct volumes <2 cm^3^ were excluded. For all the cases while omitting infarct volumes <2 cm^3^, this detection accuracy lowered to 95.7%. For any case, detection accuracy further reduced to 83.2%. Early detection accuracy (≤3 h) was 78.4% and this accuracy increased with the raise of the time after the onset of stroke symptoms from 78.4% (≤3 h) to 80.1% (3< to ≤ 8 h) to 87.9% (8< to ≤ 72 h). In addition, the SIM method also detected all 21 early ischemic infarcts (of which 15 were overlooked by stroke neuroradiologists).

### Modified SIM-based method

The promising results obtained for early ischemic stroke detection encouraged us to further study the SIM to improve its performance for hyperacute ischemic stroke. Two quantities were studied in the SIM formula: (1) parameter selection and their value setting as well as (2) its components (Gomolka et al., 2016).

In the standard (original) SIM method the P-ratio was sampled with six density ranges and the N-ratio with nine density ranges giving rise to 54 parameter combinations. This parameter setting resulted in quick infarct detection and localization in 7 s. The modified SIM method employed a wider spectrum of density ranges in terms of their span (from five to 40 HU), coverage, and numbers, including finer ranges, resulting in a total of 168 parameter combinations.

These parameters were modified and examined only in the P-ratio as the study showed that the N-ratio was optimally formulated and the MDV was excluded from the modified SIM for efficiency as its average value was similar in the infarcted and normal hemispheres among all the early scans (indicating that early infarction causes changes in density distribution rather than forming hypodense areas).

As previously reported in Gomolka et al. (2016) the modified SIM method was evaluated on 70 early (for the detection) and 70 follow-up (to set of the gold standard) ischemic stroke scans from two centers. The best performance was obtained for the P-ratio including seven percentile subranges within the range of 35th-75th percentile achieving a 76% ischemic hemisphere detection rate and 54% sensitivity in spatial localization of hyperacute ischemia.

### AI-based methods

Artificial Intelligence (AI) is defined in the Merriam-Webster dictionary (https://www.merriam-webster.com/) as: “(1) a branch of computer science dealing with the simulation of intelligent behavior in computers, and (2) the capability of a machine to imitate intelligent human behavior”. Machine learning is one form of artificial intelligence that “is devoted to building algorithms that allow computers to develop new behaviors based on experience” (https://www.merriam-webster.com/). In other words, machine learning develops algorithms enabling computers to learn from existing data without explicit programming.

Machine learning methods are subdivided into supervised learning and unsupervised learning. In supervised learning, the algorithms are first trained by employing some existing “gold standard” or “ground truth”; in the considered application this is a collection of brain NCCT scans classified into infarcted versus no infarcted. Supervised learning methods include, among others, linear regression, support vector machines, decision trees, random decision forests, and k-nearest neighbors (k-NN) algorithm (classifiers for ischemic stroke lesion segmentation are reviewed and compared by Maier et al. (2015)). In contrast, unsupervised learning attempts to discover previously unknown classes, patterns, and/or structures in the data with no given classification nor previous training. Unsupervised learning methods include, among others, k-means clustering, mixture models, and hidden Markov model. In radiology at present, the dominant type of machine learning algorithm is the artificial neural network (ANN) which is a cluster of interconnected nodes (Dreyer & Geis, 2017). An ANN with multiple layers of interconnected nodes with representation learning is termed deep learning. Deep learning has recently become the principal form of machine learning because of a convergence of theoretic advancements, openly available computer software, and hardware with adequate computational power (Zaharchuk et al., 2018). Deep learning through computationally efficient convolutional neural networks (CNNs) is well-suited for imaging (Zaharchuk et al., 2018). The CNNs require a large amount of training data to avoid overfitting, and once the network parameters have converged an additional training step is performed to fine-tune the network weights. To reduce the amount of training data and to produce more precise segmentation of biomedical images, U-net architecture is developed based on CNNs (Ronneberger, Fischer & Brox, 2015).

Rajini & Bhavani (2013) proposed a detection method by employing texture features combined with various machine learning methods. The method consists of five stages: preprocessing, segmentation, brain midline tracing, extraction of 14 texture features (using a gray level co-occurrence matrix between the left and right hemispheres), and classification (by a binary classifier). As reported earlier by Rajini & Bhavani (2013) the method was validated quantitatively on 15 ischemic cases and, to distinguish an ischemic from normal hemisphere by applying a support vector machine, k-nearest neighbors, artificial neural network, and decision tree classifiers, it achieved the accuracy of 98%, 97%, 96%, and 92%, respectively.

Sales Barros et al. (2019) proposed an infarct segmentation method utilizing CNN deep learning. The goal was to segment an infarct to calculate its volume in follow-up NCCT scans acquired between 12 h and 2 weeks after stroke onset. The method has two steps as previously described in Sales Barros et al. (2019): preprocessing to segment the intracranial region (through thresholding, region growing, and morphological operations) and CNN-based infarct segmentation (with the CNN architecture with two convolutional layers followed by two fully connected dense layers, each dense layer with 256 nodes).

For validation 396 NCCT stroke scans were employed to test segmentation performance, and additional 570 scans for training, and 60 for parameter fine-tuning. Patients with anterior circulation stroke were selected for this study, which is its major limitation. A single trained CNN achieved for all tested 396 patients the DSC of 18%. As this value is low, the scans were additionally divided into three infarction classes with fixed thresholds: severe of [14, 22] HU, intermediate of [22, 32] HU, and subtle of [32, 44] HU. Then by employing three CNNs, the corresponding values of the DSC were 78% for the severe class with 67 cases, 61% for the intermediate with 204 cases, and 37% for the subtle class with 125 cases.

Kuang et al. (2019) proposed a deep learning method to automate the ASPECTS score based on texture features extracted from each ASPECTS region to subsequently train a random forest (RF) classifier. The classifier was trained for 157 cases. The method tested on 100 patients resulted in a sensitivity of 66.2%, specificity of 91.8%, and area under the curve of 0.79. This performance was further improved when the ASPECTS was dichotomized (>4 and ≤4) achieving a sensitivity of 97.8%, specificity of 80%, and area under the curve of 0.89.

Three deep learning approaches have been proposed by the group of Kuang, Menon, and Qiu to segment follow-up NCCT scans to measure post-treatment cerebral infarct volumes for evaluating the effectiveness of endovascular therapy of acute ischemic stroke patients.

Kuang, Menon & Qiu (2019a) presented an infarct segmentation method that combines machine learning exploiting cascaded RF and interactive segmentation. The method includes three major steps as reported by Kuang, Menon & Qiu (2019a): expert initialization, RF learning and classification (with a two-stage training and testing classifier), and convex optimization-based segmentation. The initialization step requires the user’s input knowledge to pre-label some voxels in the infarcted region and background on a few axial slices aiming to lessen the detected false positives (making in this way the method semi-automated). A cascaded RF learning is applied to classify each voxel into normal or ischemic, and to calculate an infarct probability map. Four kinds of features are extracted: intensity, statistical information in the local region, the symmetric difference compared to the contralateral side (by using image symmetry), and the spatial probability of infarct occurrence. These features are input into the RF to train a first-stage classifier whose coarse results of segmentation are employed to train a second-stage fine classifier with fivefold cross-validation. The RF estimated infarct probability map calculated by the second-stage classifier with user input knowledge is subsequently included in a convex optimization function to get the final segmentation. One hundred stroke patients were used in this study, of which 70 scans for evaluation and 30 for training achieving the DSC of 79%. The method considerably outperformed some other AI methods, including the RF-based methods and CNN-based U-net.

Another method proposed by Kuang, Menon & Qiu (2019b) is based on dense Multi-Path Contextual Generative Adversarial Network (MPC-GAN). It makes use of a dense multi-path U-Net as a generator regularized by a discriminator network. The generator and discriminator input contextual information, such as bilateral intensity difference, infarct location probability, and distance to CSF. The MPC-GAN network was trained on 60 patients, fine-tuned on 10 patients, and 30 patients were used for validation yielding the DSC of 72.6%. The MPC-GAN method outperformed some state-of-the-art segmentation methods, such as the U-Net, U-Net based GAN, and RF-based segmentation method.

Kuang, Menon & Qiu (2019c) presented a deep learning-based semi-D-net method for the simultaneous segmentation of infarcts and hemorrhages. The method integrates network learned semantic information, local image context, and user initialized prior to a multi-region contour evolution scheme, which subsequently is globally optimized by a convex relaxation technique. The method also introduces a D-Unet architecture that follows that of U-Net, and semi-D-Unet that additionally requires user input knowledge. As reported earlier by Kuang, Menon & Qiu (2019c) a quantitative evaluation using 30 cases yielded the mean DSCs of 67.4% for ischemic infarct, 65.3% for hemorrhage, and 72.5% for both. Post-processing using multi-region evolution and the introduction of user interactions greatly improved accuracy. The proposed method outperforms other deep-learning methods including the U-net, D-net, demi RF, and semi-U-net.

## Discussion

The discussion covers a comparison of the individual methods and evaluation of their advantages and limitations, a characterization of the groups of the methods, a proposal of a new classification of the methods, and as a conclusion a recommendation for future development.

The proposed methods are usually a combination of diverse techniques and approaches, and the main approaches are various image processing and analysis techniques, assessment of asymmetry between the left and right cerebral hemispheres, ROI-based analysis, and AI-based classifiers.

The reviewed methods are summarized and compared in Table 1 taking into account: author(s), type of method along with techniques, use of brain symmetry (with respect to the image or by calculating the MSP), ROI-based analysis (along with the number of employed ROIs), type of stroke/infarct (ischemic, hemorrhagic, hyperacute ischemic, acute ischemic, chronic ischemic, follow-up ischemic, lacunar, infarct with hemorrhagic transformation, and infarct with leukoaraiosis), and validation (including the number of stroke cases and availability of quantitative validation).

**Table 1:** Comparison of automated methods for ischemic infarct detection, localization , and segmentation in NCCT scans of the human brain.

Author(s)	Method (group and techniques)	Left/right symmetry		ROI analysis		Type of stroke	Validation	
		Y/N	MSP	Y/N	Number of ROIs		Number of stroke cases	Quantitative assessment
Matesin, Loncaric & Petravic (2001)	IPA; rule-based, region growing	Y	N	N	NA	I	NA	N
Meilunas et al. (2003)	IPA; contour-based, filtering, smoothing	N	N	N	NA	I	NA	N
Usinskas et al. (2003)	IPA; mean, standard deviation, histogram, co-occurrence matrix	N	N	N	NA	I	NA	N
Usinskas, Dobrovolskis & Tomandl (2004)	IPA; texture-based, thresholding (not automated)	N	N	N	NA	I	NA	N
Przelaskowski et al. (2007)	IPA; wavelet-based	N	N	N	NA	I	30	Y
Chawla et al. (2009)	IPA; intensity-based, wavelet-based, two-level classification	Y	N	N	NA	I-a, I-ch, H	9	Y
Tang, Ng & Chow (2011); Tang, Ng & Chow (2013)	IPA; texture analysis, radius variable ROIs	Y	N	Y	Variable	I-a, I-ch	10 acute, 10 chronic	Y
Boers et al. (2013)	IPA; region-growing with multiple thresholds	N	Y	N	NA	I-fu	34	Y
Vos et al. (2013)	IPA: Bayes classification, marching cubes segmentation, supervised classification	Y	NA	N	NA	I	NA	Y
Tyan et al. (2014)	IPA; edge detection, region growing, blurring	Y	N	Y	8	I	26	Y
Ray & Bandyopadhyay (2016)	IPA; textural analysis, watersheding, thresholding	Y	N	Y	4	I	0	N
Maldijan et al. (2001)	BA; limited to MCA, interpolation, normalization	Y	N	Y	2	I	15	Y
Nowinski (2020b)	BA; two atlases, atlas individualization, ventricular system extraction, statistical tests	Y	Y	Y	Many	I, H	Several	Y (component algorithms)
Gillebert, Humphreys & Mantini (2014)	IT; normalization, smoothing, statistical analysis	N	N	N	N	I, H	24	Y
Nowinski et al. (2013)	SIM; original, CSF/WM/GM calculation, density sampling	Y	Y	N	NA	I-ha, I-a, I-ht, I-ch, I-lac, I+la	576	Y
Gomolka et al. (2016)	SIM; modified, CSF/WM/GM calculation, density sampling	Y	Y	N	NA	I-ha	70	Y
Rajini & Bhavani (2013)	AI; texture features, classifiers (support vector machine, k-nearest neighbors, artificial neural network and decision tree)	Y	N	N	NA	I	15	Y
Sales Barros et al. (2019)	AI; segmentation, region growing, morphological operations, deep learning with CNN	N	N	N	NA	I-fu	396 (67, 204, 125 for individual classes)	Y
Kuang et al. (2019a)	AI; texture features and RF classifier for ASPECTS	N	N	Y	10	I	100	Y
Kuang, Menon & Qiu (2019a)	AI; cascaded RF with interactive segmentation	Y	N	N	NA	I-fu	70	Y
Kuang, Menon & Qiu (2019b)	AI; dense MPC-GAN	N	N	N	NA	I-fu	30	Y
Kuang, Menon & Qiu (2019c)	AI; semi-D-net with user initialized prior	N	N	N	NA	I-fu, H	30	Y

**Notes.**

AI AI-based method BA brain atlas-based method CCN AI convolutional neural networks H hemorrhagic stroke I ischemic stroke I-a ischemic stroke, acute infarct I-ch ischemic stroke, chronic infarct I-fu late ischemic stroke, follow-up images I-ha ischemic stroke, hyperacute infarct I-ht ischemic stroke, infarct with hemorrhagic transformation I-lac ischemic stroke, lacunar infarct I+la ischemic stroke, infarct with leukoaraiosis IPA image processing and analysis-based method IT intensity template-based method MCA middle cerebral artery territory MPC-GAN dense Multi-Path Contextual Generative Adversarial Network MSP midsagittal plane calculated in 3D NA non- available/applicable ROI region of interest processing RF AI random forest SIM stroke imaging marker-based method Y/N yes/no

### Characterization of groups and evaluation of methods

**Figure 1:**
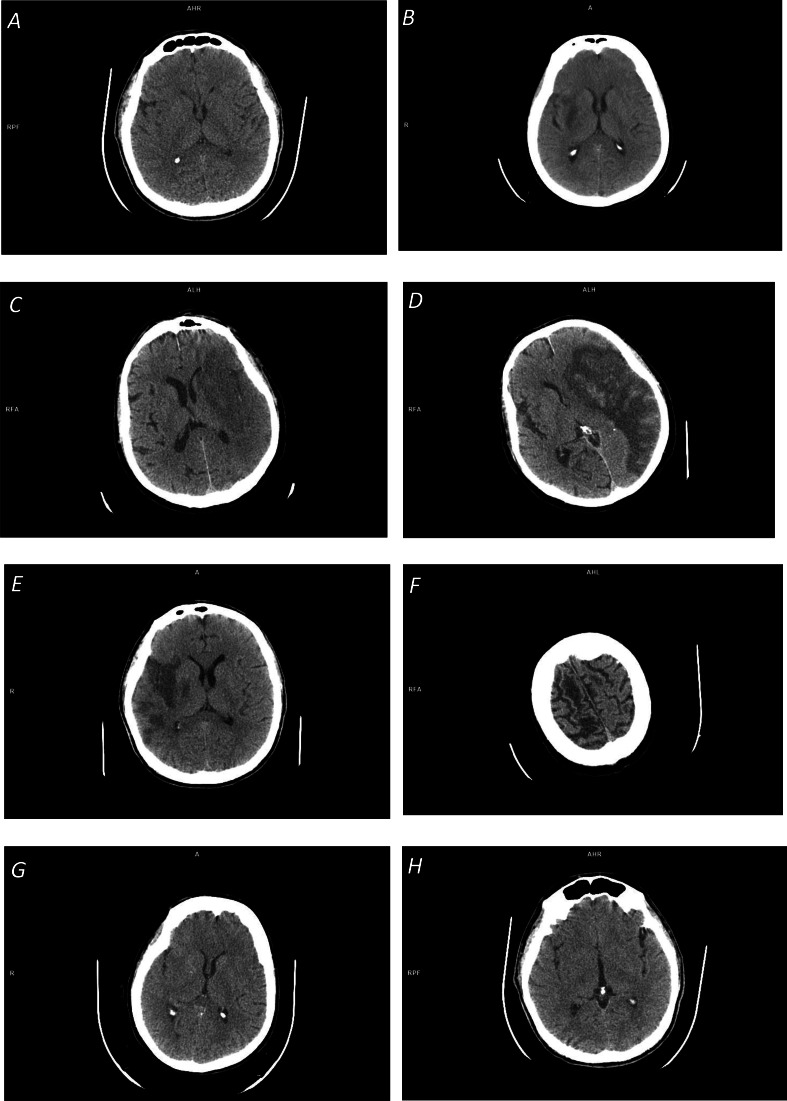
Illustration of space-dependent ischemic stroke changes on NCCT scans: (A) Early ischemic stroke with invisible changes; (B) focal inhomogeneous ischemic lesion in the right basal ganglia with a fuzzy border; (C) focal large ischemic presence in the left MCA territory with a partly fuzzy ischemic lesion at its posterior border; (D) focal large ischemic presence in the left MCA territory with a clear ischemic lesion border (along with hemorrhagic transformation); (E) multi-focal ischemic lesion of the right MCA territory; (F) overlapping density cum a partial volume effect in cerebrospinal fluid spaces; (G) distributed ischemic presence, obscuring of basal ganglia density (in the right lentiform nucleus) and loss of distinction between gray and white matter; and (H) distributed early ischemic presence, sulcal effacement in the right fronto-parietal region.

The reviewed methods enable automated detection, localization, and/or segmentation of ischemic lesions. An advantage of having the infarct segmented is that it then can easily be quantified, including calculation of its volume, which is an important radiologic outcome measure of the effectiveness of endovascular therapy. However, ischemic infarcts on NCCT images show neither homogenous density nor sharp edges, suffer from a low signal to noise ratio, and interfere with chronic infarcts and leukoaraiosis, among others, as illustrated in Fig. 1. Therefore, their accurate segmentation is difficult for real clinical data, if possible at all for acute and hyperacute cases. A more practical approach seems to provide infarct detection cum localization with the infarct volume estimated statistically by applying, for instance, a method presented by Nowinski et al. (2013). Alternatively, infarct segmentation is carried out on follow-up NCCT images as presented by Sales Barros et al. (2019), Kuang, Menon & Qiu (2019a), Kuang, Menon & Qiu (2019b) and Kuang, Menon & Qiu (2019c)).

The majority of the reviewed methods are based on image processing and analysis. Some of these methods apply thresholding, edge detection, and region growing for infarct segmentation, however, the outcome of these operations is questionable (particularly for real clinical data). First, these operations are sensitive to noise. Second, region growing is not able to bridge gaps between multiple infarcted areas (Boers et al., 2013), see Fig. 1E. Third, the ischemic lesions are not regions with uniform intensities (see Figs. 1B, 1C) but show smaller decreases on the borders and larger decreases towards the center of the lesion (Rekik et al., 2012), so these methods are not able to clearly determine the lesion boundary. And fourth, the density range of ischemic lesions overlaps with that of CSF (see Fig. 1F), and because of this and a partial volume effect in the area around CSF, the latter may be falsely included in the segmented region.

Several methods employ smoothing as a preprocessing step, which may have an impact on a precise delineation of lesion boundaries (Seghier et al., 2008). Moreover, larger smoothing values penalize the detection of small lesions by blending them with the surrounding tissues.

To cope with some of these shortcomings, more powerful approaches have been proposed in image and wavelet domains with a battery of operations, a plurality of texture attributes (such as energy, entropy, moments, inertia, shade, prominence, correlation, skewness, kurtosis, and variance), and diverse classifiers. These approaches are enhanced by a left–right hemisphere comparison and ROI processing and analysis.

It also shall be noted that some authors claim their methods to be automated, but they are actually semi-automated requiring human interaction, such as a seed point placement (Boers et al., 2013) or a threshold setting (Usinskas, Dobrovolskis & Tomandl, 2004).

The brain atlases are useful means for image partitioning by the individualized atlas (or atlases) superimposed on a scan, enabling in this way an ROI-based analysis and a comparison of the right and left cerebral hemispheres (Nowinski, 2020a). The number of image partitions varies from a few for some part of the brain (like only two in the MCA territory (Maldijan et al., 2001)) to many delineated by multiple complementary whole-brain atlases (Nowinski, 2020b). A fast and automated atlas-to-scan mapping is critical in stroke image management and we have developed earlier suitable methods for this purpose (Nowinski et al., 2006a; Volkau et al., 2012).

The intensity template based-methods exploit a common approach for abnormality detection by comparing a patient brain to some reference, neurologically normal control brain. To enable comparison with a plurality of control brains, an intensity template must be created by normalizing them to the same stereotactic space. Then, the stroke patient scan is normalized to the template and statistically compared to it on a voxel-by-voxel basis aiming to identify typical areas. In contrast to brain atlas-based methods, this group of methods can only be employed to stroke patient scans that have the same image modality as the intensity template. In the case of NCCT, the patient scan-template comparison aims at identifying regions with hypo- or hyper-intensity indicating a suspected ischemic or hemorrhagic lesion, respectively. An advantage of this approach is that it handles simultaneously both ischemic and hemorrhagic stroke; in fact, Gillebert, Humphreys & Mantini (2014) claim that their intensity template-based method was more specific in distinguishing hemorrhage than ischemia. A limitation of this approach is that ischemic lesions, as discussed above, do not demonstrate uniform intensities and overlap with other regions. The same holds for hemorrhagic lesions whose properties were studied on 289 NCCT hemorrhagic stroke scans by Nowinski et al. (2014a). These lesions span a 25–88 HU range, in contrast to other studies compared in (Nowinski et al., 2014a) indicating a much narrower range, such as 60-80 HU (New & Aronow, 1976). Moreover, hemorrhagic lesions substantially overlap with GM and to some extent with WM density ranges.

Knowledge aggregation, limited to averaging of normal brain scans in the intensity template-based methods, has a much wider potential in atlas-assisted approaches. We have created a probabilistic stroke atlas aggregating radiologic (imaging) and neurologic (numerous parameters) knowledge in the population (Nowinski et al., 2014b). The atlas originally employed for the prediction of ischemic stroke outcomes (by mapping imaging into neurologic parameters) can be potentially employed to enhance lesion detection (by applying a reverse mapping).

The image processing and analysis-based and the intensity template-based methods exploit local changes caused by an ischemic lesion, while the brain atlas-based methods examine the changes occurring in the atlas-defined ROIs. In contrast to these three groups of methods, the SIM-based methods capture the patient-specific density distribution changes globally, both in the infarct itself and the surrounding it parenchyma. This feature is especially beneficial when the infarct is in the hyperacute stage, so when its focal hypodensity (see Fig. 1A) and/or any distributed presence (see Figs. 1G,  1H) might be very subtle or even hardly discerned by the human eye. The modified approach has demonstrated that the finer density sampling with a larger number of density sub-ranges improves infarct detection in the hyperacute stage. This study also confirmed that the average density of the normal and infarcted hemisphere for hyperacute cases are the same, indicating that the hyperacute stroke detection approaches based on ischemic lesion features themselves will probably fail.

As previously reported by Nowinski et al. (2013) the usage of two different infarct localization 3D bounding boxes that are superimposed on the processed scan accumulates advantages of the high sensitivity of the outer localization bounding box and the high specificity of the inner localization bounding box. Consequently, the inner localization bounding box marks the infarcted region (meaning it works like a cursor) and the outer localization bounding box estimates the infarct extent. It is worth noting that this approach can estimate the volume of an ischemic infarct.

AI has been changing our world in many aspects, and its impact will inevitably grow in the years to come. In particular, deep learning has shown remarkable promise in solving many problems in computer vision, natural language processing, and robotics (LeCun, Bengio & Hinton, 2015) with various neural network architectures proposed, including U-Net, D-Unet, ReLU, ConvNet, ResNet, ConsNet, and MPC-GAN. Neural networks are a very intensive area of research. For instance, on Google Scholar under term “neural network” there are 2.7 million results and under “convolutional neural network” 456 thousand results. More specifically, under “stroke convolutional neural network” there 25.4 thousands results, and under “acute ischemic stroke convolutional neural networks” 18.7 thousand results. In particular in stroke management, Feng et al. (2018) claim that deep learning techniques, because of their speed and power, will become an increasingly standard tool for stroke experts. Furthermore, Maier et al. (2015) compared several classification methods for ischemic stroke lesion segmentation, although for MR scans, and concluded that high-level machine learning techniques, such as CNNs and random decision forests, lead to significantly better segmentation results compared to the rather simple classification methods such as kNN, Gaussian naive Bayes, and generalized linear models.

The papers reviewed here , however, do not demonstrate the fulfillment of these promises in the considered area yet. The method by Rajini & Bhavani (2013) employs heavy image processing components combined with several simple classification methods tested on a low number of 15 patients.

The methods proposed in Sales Barros et al. (2019), Kuang, Menon & Qiu (2019a) Kuang, Menon & Qiu (2019b) and Kuang, Menon & Qiu (2019c) use advanced deep learning techniques for follow-up NCCT scans which are easier for processing than hyper-acute and acute cases. The CNN-based method by Sales Barros et al. (2019) tested on a large dataset of 396 cases yielded the DSC of 34%. For the late cases, the DSC was increased to 78%. This approach uses fixed thresholds for density ranges, in contrast to the SIM-based methods that employ patient-specific density ranges for the calculated CSF, WM, and GM.

The methods proposed by Kuang, Menon & Qiu (2019a), Kuang, Menon & Qiu (2019b) and Kuang, Menon & Qiu, (2019c) employ various network architectures tested on a relatively small number of cases yielding a moderate performance. This performance was substantially improved by introducing high-level human knowledge to drive the segmentation (which makes the methods semi-automated).

The existing powerful deep learning techniques are inferior to the SIM-based methods (even when tested on easier data). The reason for this is that the SIM-based methods capture and process the overall changes in both the infarcted region and the parenchyma for the entire density spectrum along with the employment of patient-specific ranges, while the deep learning methods seem to focus on learning the properties of the infarcted regions only and often use fixed values of parametres.

It shall be noted that the AI methods can be used optionally with preprocessing (such as (Rajini & Bhavani, 2013) to extract texture features before supervised classification) and/or with postprocessing (such as Kuang, Menon & Qiu (2019c) to perform multi-region evolution and to do image median filtering for noise elimination in segmentation of stroke lesions from CT perfusion images (Liu et al., 2019)). Preprocessing, postprocessing, and high-level domain knowledge greatly improve the accuracy of AI-based methods as illustrated by Rajini & Bhavani (2013) and Kuang, Menon & Qiu (2019c).

Most of the image processing and analysis-based methods and the brain atlas-based methods exploit the comparison of values in the left and right whole hemispheres or some parts of them, usually the MCA territories. This spatial left–right correspondence is obtained in various ways, namely, by image division into quadrants (Ray & Bandyopadhyay, 2016), using image symmetry (Chawla et al., 2009), through individualized atlas (Nowinski, 2020b), brain midline tracing (Rajini & Bhavani, 2013), or by automated calculation of the MSP in 3D (Nowinski et al., 2013). Approaches based on image symmetry or ROI reflection (Tang, Ng & Chow, 2013) will not be able to handle real clinical cases as the left–right hemisphere symmetry is generally absent in clinical stroke scans. Moreover, standard algorithms for calculation of the MSP may often fail for some stroke NCCT scans acquired in the emergency room due to a large brain tilt. Therefore, we have developed a dedicated algorithm for MSP calculation to robustly handle stroke cases (Puspitasari et al., 2009).

Some of the image processing and analysis-based and the brain atlas-based methods employ an ROI analysis. The number of ROIs varies across methods, namely, 2 (Maldijan et al., 2001), 4 (Ray & Bandyopadhyay, 2016), 8 (Tyan et al., 2014), 10 (ASPECTS), and multiple when generated by brain atlases (Nowinski, 2020b). The number of ROIs per method may be fixed (as in the abovementioned methods) or be variable as in (Tang, Ng & Chow, 2013). The shape of ROIs is predefined by the way of image partitioning (Tyan et al., 2014; Ray & Bandyopadhyay, 2016), results from the constructed brain atlases (anatomical structures and vascular territories) or is given (e.g., circular ROIs in Tang, Ng & Chow (2013). The size of ROIs during processing is mostly fixed or variable as in Tang, Ng & Chow (2013) being circular with an adjustable radius.

The scope of validation varies among the reviewed methods, with some with no quantitative validation at all and the majority of them with a small number of stroke cases. Until today to our best knowledge, the standard SIM method by Nowinski et al. (2013) tested quantitatively on 576 stroke cases from four centers in two countries is the most thoroughly validated method in terms of the number of stroke cases and their variety. The most highly validated method of the AI-based group is that of Sales Barros et al. (2019) with 396 cases, though resulting in a very low performance (the DSC of 18%). The highest number of proposed methods belong to the image processing and analysis-based group, and the most highly validated method of this group used 30 cases (Przelaskowski et al., 2007).

Finally, the reviewed methods differ in their novelty and intellectual property. Namely, three methods are patented, that of Tang, Ng & Chow (2011) holds one US patent (Tang, Ng & Chow, 2013), and the methods by (Nowinski et al., 2013; Nowinski, 2020b) are based on four US patents and several US patent applications pending (out of our 17 US stroke-related patents listed in Nowinski, 2020b).

This comparison of methods has limitations in terms of completeness, performance measures, task performed, criteria for method grouping, and testing scan selection. Although we have tried our best to have this review as complete as possible using PubMed and Google Scholar, there might be some relevant works not listed there. Different authors use various performance measures, which hinder a fair comparison of methods. Infarct detection and infarct segmentation are two different tasks. Infarct detection uncovers the presence of any infarcted region (possible with its localization) while infarct segmentation is performed mainly to quantify the cerebral infarct volume as an important outcome measure. Moreover, the majority of infarct segmentation methods are tested on follow-up scans, where ischemic infarcts are more prominent than in acute cases. A specific method often is a combination of various techniques, so the groups of methods may overlap and the presented grouping of them is not unique. For instance, several image processing and analysis-based methods employ classifiers, which are part of image analysis and also of AI; conversely, some AI methods employ image processing techniques for pre- and post-processing.

**Figure 2:**
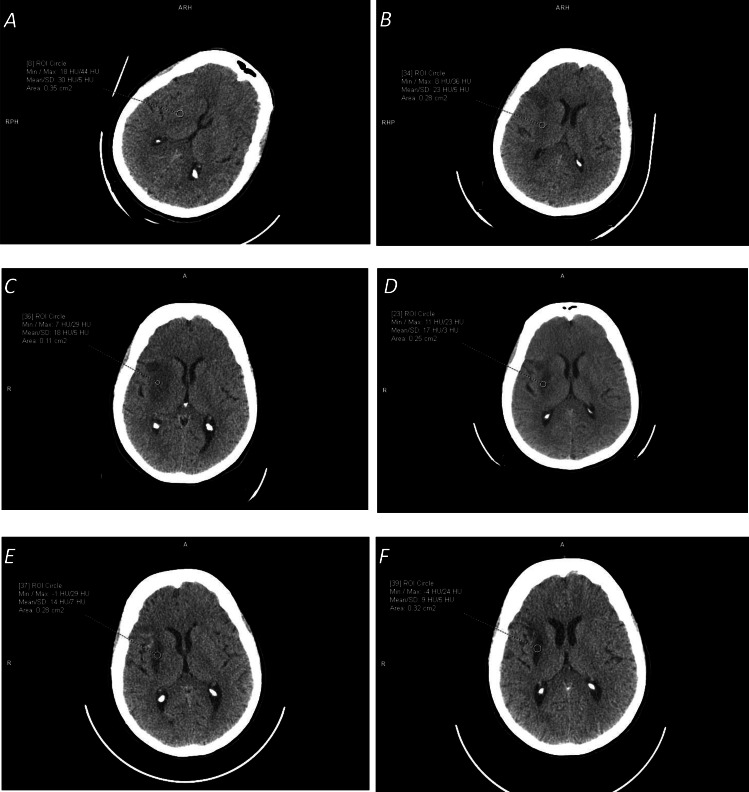
Illustration of time-dependent ischemic stroke changes in terms of appearance and Hounsfield Unit (HU) density characteristics (measured in the marked circular regions) for the same patient for the stroke onset after: (A) 1.5 hours (with [18,44] HU range); (B) 24 hours (with [8,36] HU range); (C) one week (with [7,29] HU range); (D) two and a half weeks (with [11,23] HU range); (E) one month (with [14,7] HU range); and (F) two months (with [-4,24] HU range). Note the change in the appearance of the ischemic lesion and its continuously decreasing density from the mean value of 30 HU to 9 HU.

Finally, our experience shows that probably the most critical for a fair comparison is the selection of a dataset for testing. NCCT ischemic stroke scans differ from being easy for processing to difficult; from pure ischemic stroke to stroke with hemorrhagic transformation, chronic infarct, and/or leukoaraiosis; from small lacunar infarcts to large infarcted areas; and from hyper-acute stroke (1.5 h from the stroke onset) to late stroke (several weeks after the stroke onset) to chronic infarcts (several months after the stroke onset). This difference in data selection for the same method was demonstrated, for instance, by Sales Barros et al. (2019), where the performance measured by the DSC ranged from 37% for the subtle infarct class to 78% for the severe infarct class; as well as by Nowinski et al. (2013) for the detection accuracy raising from of 78.4% for cases with ≥3 h from the stroke onset to 87.9% for cases with 8<to ≥ 72 h from the stroke onset. Time-dependent ischemic stroke changes over a two-month period in terms of appearance and HU density characteristics for the same patient are illustrated in Fig. 2. Note the growing prominence of the ischemic lesion and the decreasing mean density from 30 HU (1.5 h) to 23 HU (one day) to 18 HU (one week) to 13 HU (one month) to 10 HU (two months.)

### Classification of methods

Rekik et al. (2012) divided methods for ischemic stroke image management into four groups: pixel and voxel-based classification (the most common), image-based segmentation, atlas-based segmentation, and deformable model-based segmentation. In Section 2 we have classified the reviewed methods into five groups: image processing and analysis-based, brain atlas-based, intensity template-based, SIM-based, and AI-based. Additionally to this classification, In Table 1 some supplementary criteria are applied including a left–right hemisphere symmetry, employment of ROI-based analysis, and type of validation.

In fact, these divisions are somehow arbitrary, as the methods typically use a battery of various techniques ranging from image processing and analysis to atlas-assisted processing to statistical analysis to machine knowledge.

Therefore, we propose here another classification scheme that is more related to a strategy of ischemic infarct management than to a particular technique. Then, from a standpoint of image scope, one strategy is to provide local or regional processing and analysis to detect ischemic lesion changes, and on the other hand, the whole brain scan can be processed to detect ischemic changes. From a standpoint of image handling, the image spatial extent can be sampled and processed or its density range can be sampled. Hence, this classification can be considered as a 2 × 2 matrix with local versus global processing and analysis, and density versus spatial sampling. Then, for instance, the SIM-based methods belong to the global, density sampling category with multiple density bands; the atlas-based methods to spatial sampling category that can be regional or global; and the intensity template-based methods to the local category as it detects in the lesion area (they also may be considered as a marginal case of density sampling with a single band). The spatial sampling rate may range from low (Maldijan et al., 2001), to medium (ASPECTS), to high (Nowinski, 2020b). The density sampling rate is also variable, considered being medium in the standard SIM-based method and high in the modified SIM-based method.

## Conclusion

Future studies are necessary to develop more efficient methods and we recommend two directions for method development. One is AI, as it is considered radiology’s next frontier (Dreyer & Geis, 2017) and deep learning techniques are supposed to become a standard tool for the modern stroke specialist (Feng et al., 2018), although the results demonstrated so far in ischemic infarct detection and segmentation from NCCT using the AI methods are moderate. At present, the SIM-based methods outperform the state-of-the-art deep learning techniques, because they process the overall changes in the infarcted region and the parenchyma for the entire density spectrum with patient-specific density ranges, while the deep learning methods seem to focus on learning in infarcted regions only with fixed parameter values.

Another direction is to combine the advantages of the SIM-based method (Nowinski et al., 2013) and the multi-atlas guided method (Nowinski, 2020a) enhanced by the probabilistic stroke atlas (Nowinski et al., 2014b). In other words, the future development shall be directed toward the combination of the global methods with a high sampling both in space and density along with the employment of merged radiologic and neurologic data.

## Additional Information and Declarations

### Data Availability

The following information was supplied regarding data availability: This is a review article; there is no raw data.
